# Integrating BSA-Seq with RNA-Seq Reveals a Novel Fasciated Ear5 Mutant in Maize

**DOI:** 10.3390/ijms24021182

**Published:** 2023-01-07

**Authors:** Pengshuai Yan, Weihua Li, Enxiang Zhou, Ye Xing, Bing Li, Jing Liu, Zhanhui Zhang, Dong Ding, Zhiyuan Fu, Huiling Xie, Jihua Tang

**Affiliations:** 1 National Key Laboratory of Wheat and Maize Crop Science, College of Agronomy, Henan Agricultural University, Zhengzhou 450002, China; 2 The Shennong Laboratory, Zhengzhou 450002, China

**Keywords:** maize, fasciated ear, phytohormone, BSA-seq, RNA-seq

## Abstract

Increasing grain yield is required to meet the rapidly expanding demands for food, feed, and fuel. Inflorescence meristems are central to plant growth and development. However, the question concerning whether inflorescence development can be regulated to improve grain yield remains unclear. Here, we describe a naturally occurring single recessive mutation called *fea5* that can increase grain yield in maize. Using bulk segregant analysis sequencing (BSA-seq), the candidate region was initially mapped to a large region on chromosome 4 (4.68 Mb–11.26 Mb). Transcriptome sequencing (RNA-seq) revealed a total of 1246 differentially expressed genes (DEGs), of which 835 were up-regulated and 411 were down-regulated. Further analysis revealed the enrichment of DEGs in phytohormone signal transduction. Consistently, phytohormone profiling indicated that auxin (IAA), jasmonic acid (JA), ethylene (ETH), and cytokinin (CK) levels increased significantly, whereas the gibberellin (GA) level decreased significantly in *fea5*. By integrating BSA-seq with RNA-seq, we identified *Zm00001d048841* as the most likely candidate gene. Our results provide valuable insight into this new germplasm resource and the molecular mechanism underlying fasciated ears that produce a higher kernel row number in maize.

## 1. Introduction

Maize (*Zea mays L.*) is one of the most economically important and globally cultivated crops along with wheat and rice. Based on statistics from the Food and Agriculture Organization (FAO), total maize production has surpassed wheat and rice to reach 1 billion tons [1]. Increasing maize grain yield has long been a key target in maize breeding.

Kernel row number (KRN) is directly related to grain yield [2]. Teosinte, the wild ancestor of maize, has two rows of grain, whereas the domestication of maize resulted in modern varieties with more than eight rows [3]. KRN is initiated by the inflorescence shoot meristem. Therefore, knowledge of the genes affecting maize ear inflorescence development may lead to better grain yield modeling. A better understanding of the genetic mechanisms of maize ear inflorescence development is important for breeding maize varieties with a high grain yield.

After long-term domestication and improvement, two inflorescences with distinct morphology and function were developed in modern maize: male (tassel) and female (ear) inflorescences. Vegetative growth is transformed into reproductive growth as the apical meristem develops to form the tassel. The axillary meristem of the stem segment transforms into the female meristem followed by differentiation of the inflorescence meristems (IMs) to produce the spikelet pair meristems (SPMs). Each SPM produces two small deterministic flower meristems called spikelet meristems (SMs). The inferior flower then degenerates, and the fertile floret undergoes the development of embryos and endosperm to form grains [4]. The development and differentiation of meristems on ear inflorescences determine the number of florets on inflorescences. Therefore, ear inflorescence morphogenesis and flower development are the biological basis of maize grain formation.

At present, our understanding of the genetic regulation of maize inflorescence development mainly comes from the genetic analysis of a large number of inflorescence development mutants. Genes cloned by far are involved in the *CLAVATA-WUSCHEL* (*CLV-WUS*), *RAMOSA*, and the phytohormone regulation pathways. Genes in the CLV-WUS pathway regulate the balance between meristem cell division and differentiation to maintain the number of stem cells and continuously generate new tissue [5,6]. The genes involved in this pathway include *THICK TASSEL DWARF1* (*TD1*) [7], *FASCIATED EAR2* (*FEA2*) [8], and downstream effectors such as *FASCIATED EAR3* (*FEA3*) and *Zmfcp1* [9]. Genes involved in the RAMOSA pathway include *RAMOSAL1* (*Ra1*), *Ra2,* and *Ra3*. These genes are mainly expressed in young ear primordia of the SPM and SM [10,11,12]. 

Many hormones are known to participate in the development of maize inflorescence: auxin (IAA), gibberellic acid (GA), and cytokinins (CKs) being just a few. Genes involved in auxin synthesis and signaling include *VANISHING TASSEL2* (*VT2*) [13], *SPARSE INFLORESCENCE 1* (*SPI1*) [14], *BARREN INFLORESCENCE 1* (*BIF1*), *BIIF2*, and *BIF4* [15] among others. Loss-of-function mutations in these genes reduce the number of florets in the ear. *KNOTTED1* (*KN1*) [16] encodes a transcription factor that controls GA levels in the shoot apical meristem (SAM) by regulating expression of the gibberellin peroxidase gene *GA2OXI*. *UNBRANCHED3* (*UB3*) encodes an SBP-box transcription factor that may influence size of the IM by negatively regulating CK levels in young panicles [17]. These studies demonstrate that maize inflorescence development involves the coordinated regulation of multiple genes, and mutants with incomplete inflorescence development have potential breeding value.

In this study, we obtained a novel maize mutant called *fasciated ear5* (*fea5*). Compared with normal plants, the ears of *fea5* are thick and flat with disordered rows, and the ear diameter and kernel weight are significantly increased. We combined BSA-seq-based mapping and RNA-seq profiling to identify causal candidate genes associated with fasciated ears in maize. The results indicate that *Zm00001d048841* is the potential candidate gene, and it encodes a phosphatidylcholine 2-acylhydrolase protein that is involved in the phospholipase pathway. We hypothesize that reduced expression of *Zm00001d048841* in *fea5* disrupts phytohormone signal transduction to produce fasciated ears.

## 2. Results

### 2.1. Phenotypic Analysis of The fea5 Mutant 

**Figure 1:**
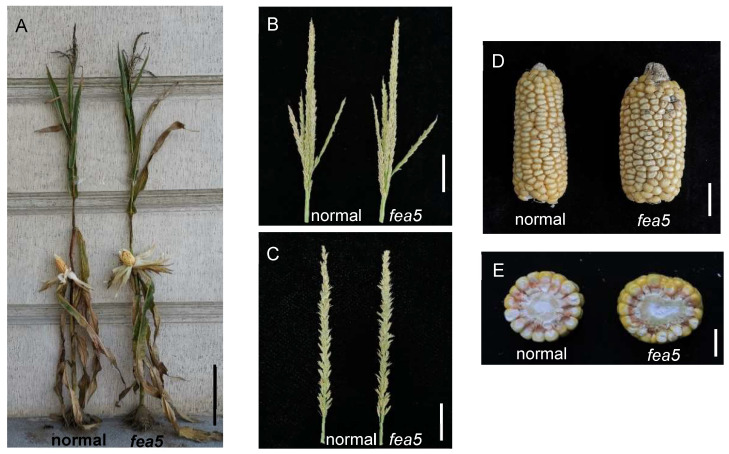
Phenotypes of *fea5* mutants. (**A**) The height of *fea5* mutants is similar to that of normal plants. Bar = 30 cm. (**B**) The tassel of *fea5* is similar to that of normal plants. Bar = 5 cm. (**C**) The tassel of *fea5* is similar to that of normal plants. Bar = 5 cm. (**D**) *fea5* ears were massively fasciated compared with normal ears and had disorganized rows. Bar = 20 mm. (**E**) Cross section of *fea5* ears. Bar = 10 mm.

The *fea5* mutant was originally discovered as a naturally occurring mutation from a Chinese elite inbred line, Lx9801. When compared with normal-ear plants Lx9801, the *fea5* mutant showed no change in vegetative plant architecture (Figure 1A) or tassel development (Figure 1B,C). *fea5* ears were massively flattened and fasciated with more numerous and irregular KRNs compared with those of normal ears (Figure 1D,E). As for other agronomic traits, ear kernel weight and ear diameter were significantly increased in the *fea5* mutant. No significant difference in plant height, ear height, maize leaf number, stem diameter, tassel branch number, or ear length was observed (Table 1).

### 2.2. The fea5 Mutant Exhibits an Enlarged and Flattened Inflorescence Meristem

**Figure 2:**
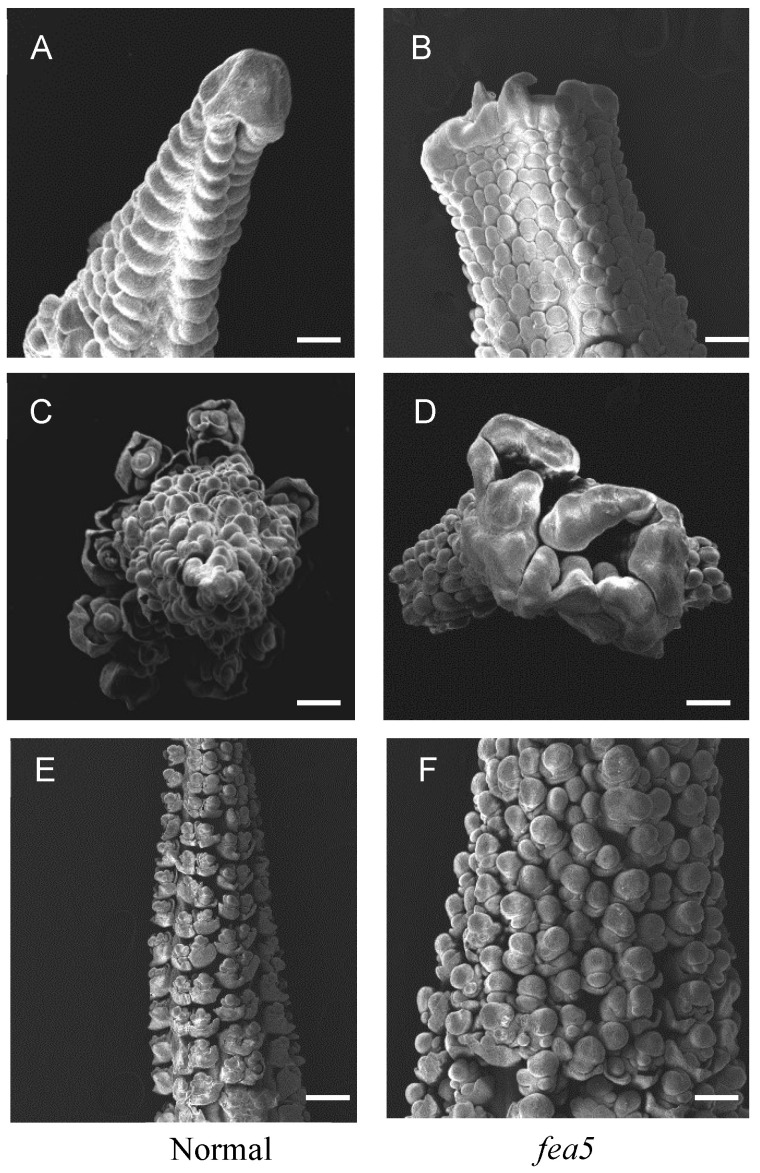
Microscopic phenotypes of *fea5* mutants. *fea5* ear IMs were enlarged and flattened in (**B**,**D**), whereas normal ears had tapered, conical shaped IMs (**A**,**C**). Bars = 500 mm. Normal ears occurred as a gradual progression and generated pairs of spikelets that remain aligned regularly (**E**), whereas in *fea5* ears, the plane of SPM branching was disrupted (**F**).

To further dissect the difference between the *fea5* and normal ears on inflorescence morphology, we observed the immature ears during development using a scanning electron microscope (SEM). When *fea5* ears were only 1–2 mm in length, the inflorescence meristems were obviously enlarged and flattened compared with normal ears (Figure 2A,B). As the developmental process continues, *fea5* ears became more fasciated, and the apical region enlarged severely compared with normal ears (Figure 2C,D). Distinct developmental stages occurred as a gradual progression in normal ears, which generated pairs of spikelets that remained aligned (Figure 2E). However, *fea5* ears developed irregular clusters of SMs due to a disruption in the plane of SPM branching (Figure 2F).

### 2.3. Whole-Genome Sequencing and BSA-Seq Analysis

The *fea5* mutant was crossed with B73, an inbred line that has a publicly-available sequenced genome, and the resulting F_1_ progeny were self-pollinated to generate a segregating F_2_ population. We randomly selected and grew some F_2_ kernels to collect and analyze 290 F_3_ ears. The ratio of normal ears (*n* = 225) to fasciated ears (*n* = 65) was approximately 3:1 (χ^2^ = 1.03 < χ^2^_0.05_ = 3.84). These results indicate that the mutant phenotype of *fea5* is caused by a single recessive mutation.

**Figure 3:**
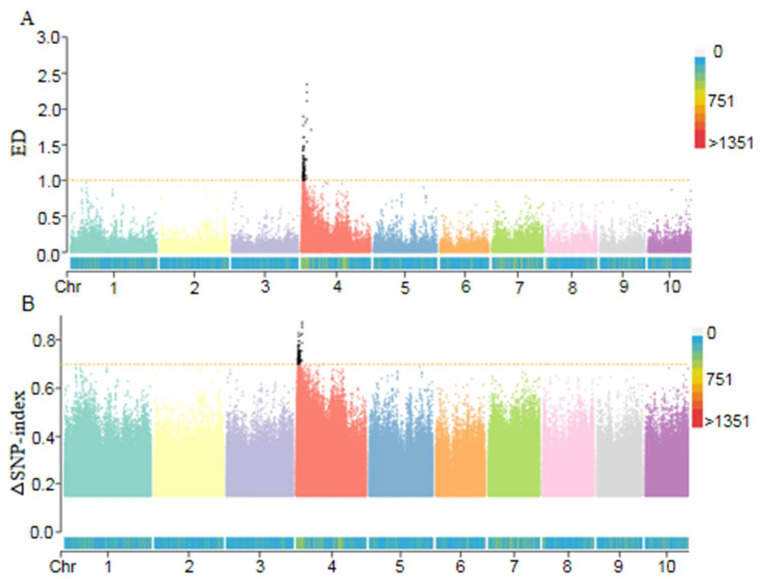
Mapping of the *fea5* mutation using BSA-seq. (**A**) ED plot of the normal and *fea5* pools. (**B**) ΔSNP-index plot of the normal and *fea5* ear pools. The orange dashed line indicates the threshold.

To identify the candidate regions underlying the *fea5* mutant, we performed preliminary mapping using BSA-seq. We pooled DNA from 50 fasciated ears and 50 normal ears from F_2:3_ mapping populations to create two pools of extreme bulked segregants. We sequenced the two extreme bulked pools and the parent *fea5* on an Illumina platform using 125 bp and 150 bp paired-end reads, respectively. Over 3.2 billion clean reads with a Q30 ratio greater than 92.48% and GC content greater than 45.96% were obtained. Of the properly-paired reads generated from *fea5* sequencing, 88.55% were mapped to the maize B73 v4 reference genome (ftp://ftp.ensemblgenomes.org/pub/plants/release−41/fasta/zea_mays/dna/, accessed on 3 January 2023). For normal-ear and fasciated-ear pools, 91.54% and 90.62%, respectively, of the reads were properly paired. The read depth of the normal-ear pool, fasciated-ear pool, and *fea5* was 44×, 71×, and 74× of the assembled reference genome, respectively (Supplementary Table S2). To identify the genomic region associated with fasciated ears, ΔSNP-index and ED analyses were performed to calculate the allele segregation of the SNPs and InDels between the normal- and fasciated-ear DNA pools. A 6.58 Mb region on Chr.4 (4.68 Mb to 11.26 Mb) was identified as the candidate region for fasciated ears based on the occurrence of significant linkage disequilibrium (Figure 3A,B). 

### 2.4. Transcriptome Profiling of the fea5 Mutant

**Figure 4:**
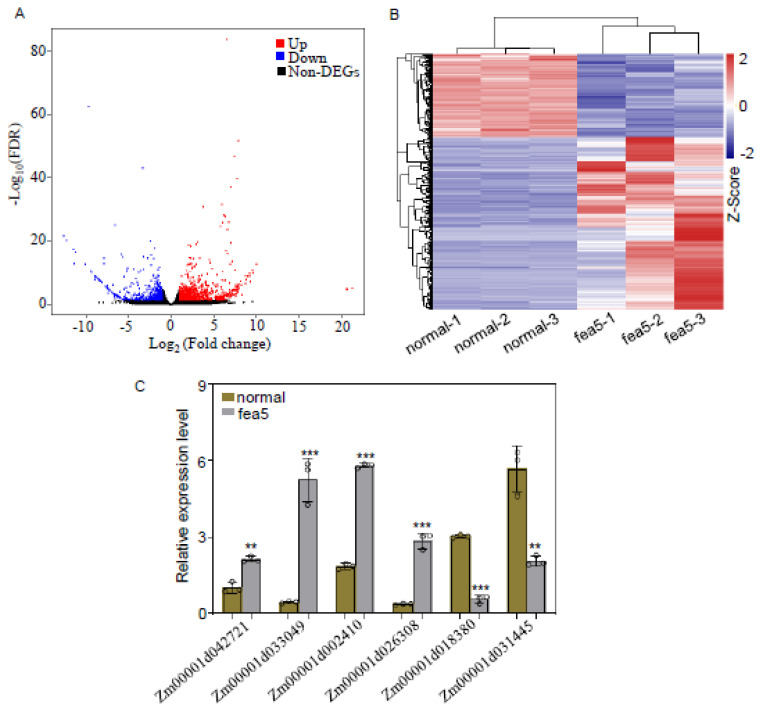
The differentially expressed genes (DEGs) between normal and *fea5* plants. (**A**) Gene expression classification of DEGs. Blue and red dots indicate down- and up-regulated genes (significance level: FDR < 0.05, Log2FoldChange >1), respectively. (**B**) Heat map of the DEGs between normal and *fea5* plants. (**C**) The selected genes were verified by RT-qPCR with three replicates per gene. Values are represented as means ± SD, ** *p* < 0.01; *** *p* < 0.001 (Student’s *t*-test).

To accelerate candidate gene identification and understand the transcriptome network underlying the phenotypic variations, RNA-seq was performed using ears at the SPM stage. After quality control and filtering of raw reads, more than 0.12 billion clean reads were available for each sample, and approximately 91% of the clean reads could be perfectly mapped to the maize B73 v4 reference genome (ftp://ftp.ensemblgenomes.org/pub/plants/release−41/fasta/zea_mays/dna/, accessed on 3 January 2023). Reads that could not be perfectly mapped to the maize genome were discarded, and only those with FPKM values higher than 0.1 were further analyzed. In addition, only genes with a log2 fold-change of ≥1 with an adjusted *p* value of ≤0.05 were further analyzed. At the SPM stage, 1246 genes were identified by DEGseq software as differentially expressed between normal and *fea5* ears. Among the DEGs, 835 were up-regulated and 411 were down-regulated in normal ears relative to *fea5* ears (Figure 4A,B). Quantitative RT-PCR (RT-qPCR) was used to verify that the expression levels of a subset of DEGs were consistent with the RNA-Seq data (Figure 4C).

**Figure 5:**
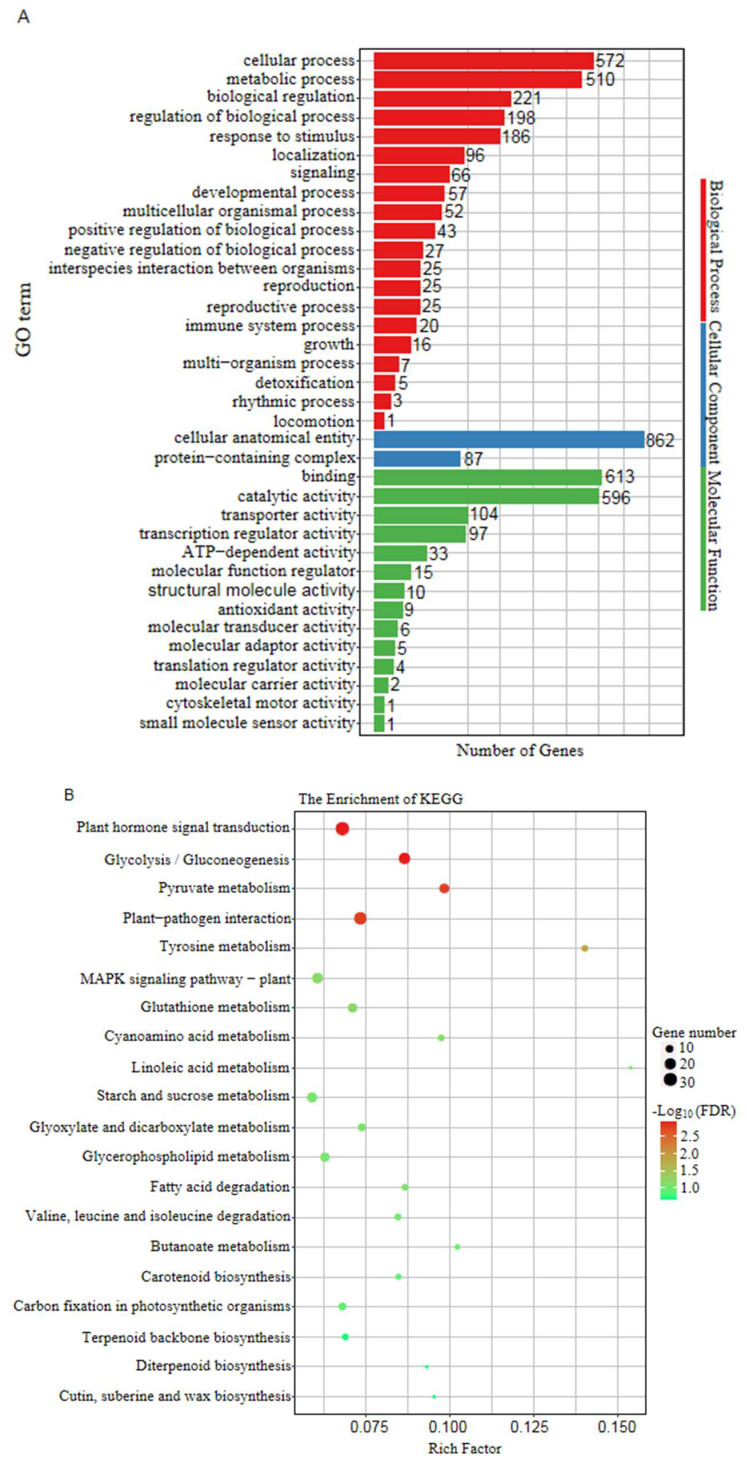
Functional analysis of the DEGs. (**A**) The GO terms in molecular function, cellular component, and biological process categories. (**B**) KEGG pathway enrichment of the DEGs. Size of the circle size is proportional to the number of genes and color denotes the range of the FDR.

Gene Ontology (GO) terms and Kyoto Encyclopedia of Genes and Genomes (KEGG) pathways were used to functionally annotate the DEGs. The DEGs could be divided into three main GO categories: biological process, molecular function, and cellular component. DEGs were enriched in 20 biological process terms, 14 molecular function terms, and 2 cellular component terms (Figure 5A). GO terms categorized under biological process mainly included the regulation of cellular process, metabolic process, regulation of biological process, signaling, developmental process, reproductive process, and immune system process. Only two cellular component terms were enriched: cellular anatomical entity and protein-containing complex. Enriched molecular function terms mainly included catalytic activity, transporter activity, transcription regulator activity, ATP-dependent activity, and structural molecule activity. KEGG analysis indicated that plant hormone signal transduction was the most enriched pathway, implicating its key role in the regulation of fasciated ears in maize. Other enriched pathways included gluconeogenesis, pyruvate metabolism, plant-pathogen interaction, tyrosine metabolism, and MAPK signaling pathway (Figure 5B). These results revealed that the expression levels of genes related to plant hormone signal transduction were disrupted in *fea5*.

### 2.5. DEGs Involved in Phytohormone Signal Transduction Are Enriched 

Based on our functional analysis of DEGs and analyses from previous studies, we focused on DEGs related to phytohormone signal transduction to unravel the molecular mechanisms governing fasciated ears in maize. As mentioned above, hormones play an important role in the regulation of inflorescence development. Consistent with this, our RNA-seq results indicated that 32 DEGs were associated with plant hormone signal transduction (Table 2). These genes were widely distributed in various hormone signaling pathways. Specifically, eight DEGs were involved in IAA signal transduction, including four down-regulated and four up-regulated genes. Eleven DEGs were involved in jasmonic acid (JA) signal transduction: two down-regulated and nine up-regulated genes. Five DEGs were involved in ethylene (ETH) signal transduction and all were down-regulated. Two up-regulated and two down-regulated genes were involved in ABA signal transduction. Two down-regulated DEGs were involved in the CK signaling pathway. The GA signaling pathway contained two DEGs, one of which was up-regulated and the other was down-regulated.

### 2.6. Phytohormones Measurement

**Figure 6:**
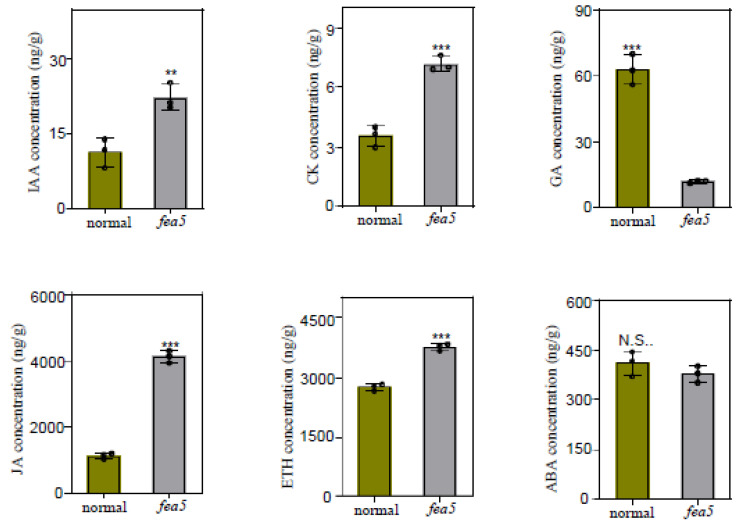
IAA, CK, GA, JA, ETH, and ABA levels in normal and *fea5* ears. Student’s *t*-test was used to compare the means ± SD. Asterisks indicate significant differences: ** *p* < 0.05 and *** *p* < 0.01. N.S.: Not significant.

To investigate changes in phytohormones between normal and *fea5* ears, we analyzed ears weighing over 0.5 g at the SPM developmental stage with each sample including three replications. The content of IAA, GA, ABA, ETH, CK, and JA was measured using HPLC-MS (Figure 6). Compared with normal ears, ETH content increased by 40% in *fea5* mutants, and the content of CK, IAA, and JA significantly increased by 1.0- to 2.6-fold. By contrast, GA content was significantly decreased by 80% in *fea5* mutants. No significant difference in ABA was detected between normal and *fea5* ears.

### 2.7. Identification of the Candidate Genes Related to fea5

**Figure 7:**
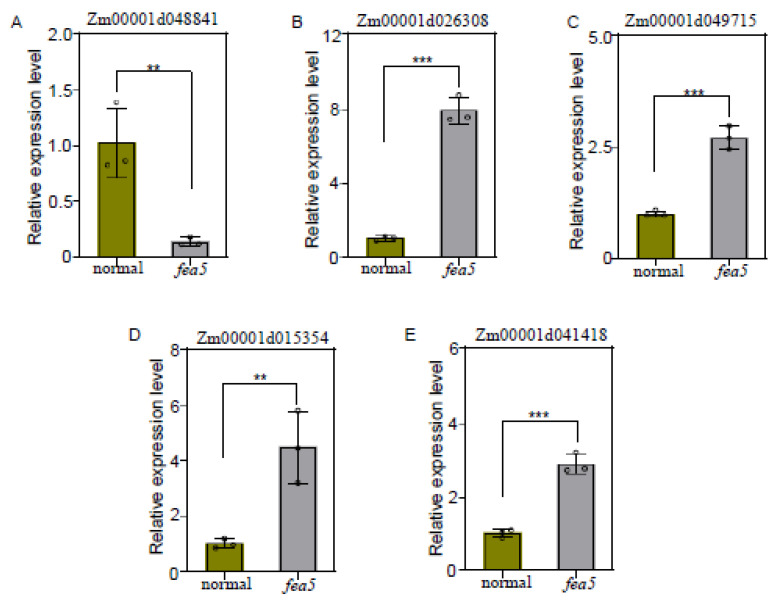
Verification of selected candidate genes in *fea5* and the normal ears by RT-qPCR. The transcript levels of *Zm00001d048841* and IAA-responsive genes *Zm00001d026308*, *Zm00001d049715*, *Zm00001d015354*, and *Zm00001d041418* were verified by RT-qPCR with three replicates per gene. Values represent means ± SD. Asterisks indicate significant differences: ** *p* < 0.01 and *** *p* < 0.001.

Within the 7 Mb mapping region, no genes related to fasciated ears had been previously reported. Our data suggested that only two genes within the confidence interval, *Zm00001d048839* and *Zm00001d048841*, were differentially expressed between normal and fasciated ears. Based on publicly available RNA-seq data (Supplementary Figure S1), *Zm00001d048841* was highly expressed primarily in the ear primordium, whereas *Zm00001d048839* had non-specific and low expression in the ear primordium. We confirmed that the expression level of *Zm00001d048841* was 4-fold lower in *fea5* using RT-qPCR (Figure 7A). Therefore, we predicted that *Zm00001d048841* might be a key gene that controls the fasciated ear phenotype in *fea5*.According to gene function predication (https://www.ncbi.nlm.nih.gov/, accessed on 3 January 2023). We found that *Zm00001d048839* encoded an unknown protein, and *Zm00001d048841* encoded a phosphatidylcholine 2-acylhydrolase protein. Zm00001d048841 may involve in the phospholipase pathway, they are required for signal transduction events during seed germination and in auxin-stimulated cell elongation [18]. The decreased *Zm00001d048841* expression level may lead to decreased phospholipase activity, which affects phytohormone signaling, resulting in fasciated ears.

### 2.8. Natural Variations in Zm00001d048841 Are Associated with KRN in Maize

**Figure 8:**
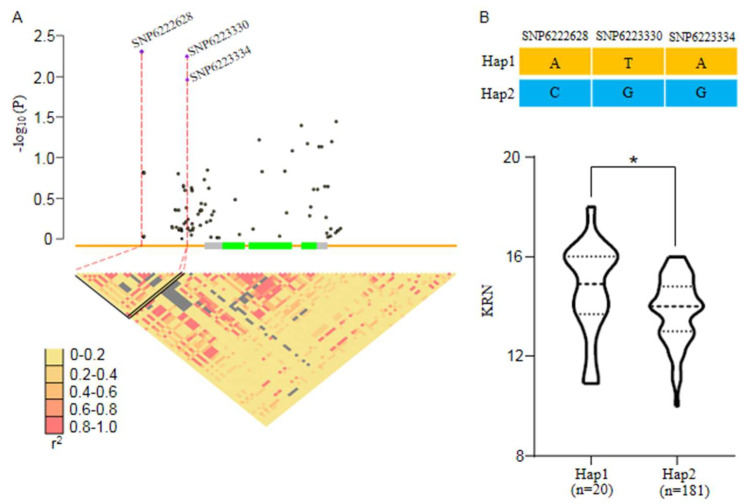
Natural variations in *Zm00001d048841* were significantly associated with maize KRN. (**A**) *Zm00001d048841*-based association mapping and pairwise LD analysis. The dots represent SNPs, and the triangles denote the lead SNP. The SNPs showing strong LD with the lead SNP are connected to the pairwise LD diagram with dotted lines and highlighted with red lines. (**B**) Haplotypes (Hap) of *Zm00001d048841* among maize natural variations. “*n*” denotes the number of genotypes belonging to each haplotype group. The KRN of each haplotype group is displayed as a violin plot. Statistical significance was determined using a two-sided *t*-test. Asterisks indicate significant differences: * *p* < 0.05.

A candidate gene association analysis was performed using data from a panel of 350 maize genotypes [19]. Three SNPs were found to be significantly associated with KRN (Figure 8A). These three SNPs were located at the promoter of *Zm00001d048841* and accounted for 7.9% of the variation in the maize KRN. In addition, SNP6222628 was in strong linkage disequilibrium (LD) with SNP6223330 and SNP6223334 (r^2^ > 0.9; Figure 8A). Two haplotype groups were defined based on these three SNPs, and the 20 inbred lines that contained haplotype1 (Hap1) had significantly higher KRNs than the 181 inbred lines that contained haplotype2 (Hap2) contained (Figure 8B). Moreover, a genetic marker could be developed based on Hap1 and used to screen for superior germplasm to provide theoretical and technical support for the genetic improvement of maize ear traits and the breeding of new varieties with increased yield.

## 3. Discussion

Fasciation patterns are very important for genetic analysis as well as improving crop yield. A better understanding of the genetics that controls fasciation could be used to better modulate crop yield while maintaining uniformity of plants and ears. Maize grain yield can be directly improved by selecting for a higher KRN in the breeding process [8]. In this study, we identified *fea5* as a novel fasciated ear mutant. We detected no differences in the cDNA sequence and expression levels of the previously reported fasciated ears-related genes *fea2* and *fea3* between normal and *fea5* plants, indicating that *fea5* is distinct from these genes (Supplementary Figure S2A–C). Based on two years of field experiments conducted in Sanya and Zhengzhou, the ear kernel weight of *fea5* mutants was 3.8–6.6% higher than normal-ear plants (Table 1). Therefore, *fea5* can serve as an excellent genetic resource for generating new high-yield maize varieties. 

### 3.1. The Combination of BSA-Seq and RNA-Seq Is an Effective Strategy for Gene Fine Mapping

BSA-seq, which uses pools of genomic DNA collected from individuals with extreme phenotypes from a segregating population, is a rapid and effective method for identifying molecular markers linked to traits and the candidate region [20]. With the rapid development of next-generation sequencing (NGS) technologies, SNPs and InDels can be quickly and easily detected in the genome. Recently, a series of NGS-based approaches have been developed: BSA-seq (or QTL-seq), bulked segregant RNA-seq (BSR-seq), MutMup, and MutMup+ [21,22,23,24]. BSA-seq has been used extensively in other taxa [25,26,27,28,29,30]. The biggest advantage of BSA-seq over other methods is its simplicity, both in terms of sample collection and data analysis. BSA-seq samples can be collected at any developmental stage and from any tissue, whereas samples of BSR-seq must be collected from specific tissues or developmental stages. In BSR-seq, allele-specific expression, as well as differential expression of genes not linked to the mutant gene in mutant versus wild-type pools, must be accounted for [31]. Hence, in this study, we collected 50 non-fasciated ears and 50 extremely fasciated ears in separate pools to conduct a BSA-seq analysis.

Integration of BSA-seq and RNA-seq is an effective strategy for gene fine-mapping [32]. To determine the candidate genes associated with fasciated ears, we performed an association analysis by combining the BSA-seq and transcriptomic data. Our results indicated that two DEGs were likely responsible for the *fea5* phenotype: *Zm00001d048839* and *Zm00001d048841*. *Zm00001d048839* was up-regulated in fasciated ears but had a low expression level, whereas *Zm00001d048841* was down-regulated in fasciated ears and had a high expression level in the ear primordium.

### 3.2. DEGs Involved in Phytohormone Pathways May Play Major Roles in the Development of Fasciated Ears in Maize

Previous studies have reported that phytohormones have major effects on ear development in maize [33]. In recent years, new roles for phytohormones in floral organ development have been discovered. Studies of the phylogenetic relationship and expression pattern of *jasmonate ZIM-domain* (*JAZ*) family genes have demonstrated that *Zea mays JAZ14* (*ZmJAZ14*) may serve as a regulatory hub for the JA, ABA, and GA signaling pathways in maize [34]. The maize *FLAVONE SYNTHASEI-1* (*ZmFNSI-1*) gene encodes the main enzyme required for the biosynthesis of flavone O-glycosides. Interestingly, *ZmFNSI-1* is expressed at a very high level in the silk, suggesting a potential role for flavone in silk development [35]. Auxin was found to be essential for the initiation of floral primordia, and the disruption of auxin biosynthesis, polar auxin transport, or auxin signaling arrested flower formation [36]. Ethylene also regulates many aspects of plant growth and development, including flower development and sex determination [37]. Mutation of *ARABIDOPSIS HISTIDINE PHOSPHOTRANSFER PROTEIN 6*, which is a negative regulator of CK signaling that expresses at the meristem flanks, caused a delay in differentiation [38].

Consistent with the above-mentioned published data, our transcriptome results revealed that many genes involved in IAA, JA, GA, CK, and ETH signaling transduction were differentially expressed between *fea5* and normal ears. For example, *Zm00001d034298*, a PIF4 transcription factor that directly activates the expression of IAA biosynthesis genes, was down-regulated by 2.16-fold in *fea5*. *Zm00001d042833* encodes the F-box protein CORONATINE INSENSITIVE1 that is essential for all jasmonate responses and is part of the SCF E3 ubiquitin ligase complex that recruits JAZ proteins for degradation by the 26S proteasome. In *fea5*, *Zm00001d042833* was down-regulated by 2.19-fold compared with the normal ears. Two DEGs involved in ETH signal transduction, *Zm00001d031445* and *Zm00001d053642* encode ETHYLENE INSENSITIVE3-LIKE (EIL)-transcription factors that activate the ETH signaling pathway. *Zm00001d031445* and *Zm00001d053642* were down-regulated by 2.22-fold and 3.41-fold in *fea5*, respectively, compared with the normal ears. *Zm00001d018178*, a bZIP-transcription factor that activates the ABA signaling pathway, was up-regulated by 3.92-fold in *fea5*. A type-B ARR transcription factor involved in plant responses to CK, *Zm00001d018380*, was down-regulated by 2 -fold in *fea5* compared with the normal ears. *Zm00001d052126*, which encodes a DELLA protein that mediates GA signaling, was down-regulated by 4.3-fold in *fea5* compared with the normal ears. Taken together, these findings indicate that phytohormone signaling may play a key role in the *fea5* mutant. 

### 3.3. Phospholipase Activity Is Involved in IAA-Stimulated Cell Division and Growth

Phytohormone signaling regulates development and communication between different regions of the SAM. As one of the most well-studied hormones, IAA drives lateral primordium initiation [33,39], and the rapid response of plant cells to IAA is mediated by the activation of phospholipases. Our RT-qPCR experiment validated four IAA responsive DGEs identified by RNA-seq, all of which were unregulated in *fea5* ears compared with the normal ears. (Figure 7B–E). Specifically, phospholipase A2 (PLA2) activity in hypocotyl segments of zucchini and sunflower causes an auxin-specific increase in growth that is partially complemented by mastoparan [40], and inhibitors of PLA2 blocked this auxin-stimulated elongation [41]. In this study, *Zm00001d048841* exhibited differential expression between normal and *fea5* ears, we therefore hypothesized that *Zm00001d048841* might be the key gene responsible for the *fea5* mutant phenotype. To test this possibility, we performed a candidate gene association analysis and detected significant differences in KRN between two groups of inbred lines that contained different haplotypes of the significant SNPs in *Zm00001d048841*. This result suggests that *Zm00001d048841* is a promising candidate gene for the *fea5* mutant phenotype. The function of *Zm00001d048841* needs to be verified and characterized by future studies.

## 4. Materials and Methods

### 4.1. Experiment Population

The maize mutant *fea5* was isolated in a screen for natural mutants during maize breeding. The normal ears Lx9801 and *fea5* were used for phenotypic evaluation, RNA-seq, and phytohormone profiling. To facilitate BSA-seq, the *fea5* mutant was crossed with B73 to generate an F_1_ population in Zhengzhou, Henan Province. The site (113.42° E, 34.48° N) is in central China and has an average annual temperature of 14.3 °C and an average annual rainfall of 640.9 mm. F_2_ individuals were obtained by selfing F_1_ plants at the Sanya Agriculture Experimental Station of the Henan Agricultural University. The site (109.20° E, 18.40° N) is located in Hainan Province. F_2:3_ individuals were obtained by selfing the F_2_ plants in Zhengzhou, Henan Province, and were used for BSA-seq.

### 4.2. Scanning Electron Microscopy (SEM)

The ears of the normal and *fea5* plants at the V12 stage (12 expanded leaves) were used for SEM observations. Normal and *fea5* ears were fixed overnight in FAA (formalin: acetic acid: 70% ethanol, 1:1:18, *v*/*v*/*v*) and dehydrated in a graded series of ethanol (70%, 80%, 95%, and 100% ethanol) and then treated with isoamyl acetate for 15 min twice to replace the remaining ethanol and subjected to critical point drying (Hitachi S-3400N). The samples were then coated with Pt particles and analyzed under a scanning electron microscope SU8020 (Hitachi, Tokyo, Japan) operating at 5 kV. 

### 4.3. DNA Library Construction and BSA-Seq Analysis

Genomic DNA was extracted from leaves using a Plant Genomic DNA Extraction Kit (product number DP305) from TIANGEN Biotech (Beijing, China) according to the manufacturer’s instructions. The F_2:3_ population generated by crossing B73 with *fea5* was used for BSA-seq. Two DNA pools were constructed by mixing equal amounts of DNA from 50 normal ears or 50 extremely fasciated ears and sequenced on an Illumina HiSeq 2000 platform. After sequencing, clean reads were obtained by removing low-quality and short reads using the Soapnuke program [42]. Clean reads were mapped to the maize B73 reference genome (ftp://ftp.ensemblgenomes.org/pub/plants/release-41/fasta/zea_mays/dna/, accessed on 3 January 2023) using BWA software with Samtools [43]. SNPs and InDels were called and filtered by removing heterozygous and missing SNPs and InDels in the pools and parental lines using GATK software [44]. The SNP index represents the ratio of reads that contained SNPs to the total number of reads [21]. The ΔSNP index is the difference between SNP indices of the bulked DNA pools. To identify candidate regions associated with fasciated ear, the ΔSNP index of each locus was calculated by subtracting the SNP index of the normal-ear pool from that of the fasciated-ear pool according to a previously described method [24]. To confirm the ΔSNP index results, a Euclidean Distance (ED) algorithm was used to identify SNPs and InDels associated with fasciated ear using the equation reported previously [45,46]. 

### 4.4. RNA-Sequencing (RNA-Seq) Analysis

Total RNA was extracted from the SPMs of normal and *fea5* ears with the TransZol Plant RNA purification kit (TransGen Biotech, China). Three micrograms of total RNA was used as input material for construction of the RNA libraries. The RNA quality and integrity were assessed using an Agilent 2100 Bioanalyzer system (Agilent Technologies, Santa Clara, CA, USA). The RNA-seq libraries were generated using the NEBNext Ultra RNA Library Prep Kit for Illumina according to the manufacturer’s instructions (New England Biolabs) and sequenced using the Illumina HiSeq 2500 Sequencing System (Berry Genomics, China). Each maize line was represented by three biological replicates. The original raw reads were generated after excluding low quality reads and adapter sequences using fastp [47]. The unique reads were then aligned to the maize B73 reference genome version 4 (ftp://ftp.ensemblgenomes.org/pub/plants/release−41/fasta/zea_mays/dna/, accessed on 3 January 2023) using HISAT2 v2.1.0 with default parameters [48]. Only perfectly matching sequences were retained for further analysis. Gene expression level was normalized by calculating the number of fragments per kilobase of transcript per million mapped reads (FPKM) [49]. Feature Counts software and the R package edgeR were used to identify the DEGs (differentially expressed genes) [50,51]. AgriGO v2.0 was used for GO (gene ontology) enrichment analysis using the maize AGPv4 reference background [52].

### 4.5. Plant Hormone Quantification

Over 2 g of tissue from normal and *fea5* ears at the SPM stage were used for plant hormone quantification using three biological replicates per line. The freeze-dried tissues were crushed in a mixer mill (30 Hz, 1 min). A total of 50 mg of powder was extracted with 1 mL of a methanol:water:formic acid (15:4:1, *v*/*v*/*v*) solution containing 0.001 ng of an internal standard. After vortexing, centrifuging, and concentrating it, the concentrated sample was dissolved in 100 μL of an 80% methanol andwater solution. Finally, each sample was filtered through a 0.22-μm microporous membrane for the high-performance liquid chromatography (HPLC) analysis. HPLC was used by Suzhou Comin Biotechnology Co. Ltd. (Suzhou, China) to quantify the phytohormones IAA, GA, ABA, JA, CK, and ETH. The hormone content was normalized by freeze-dried mass.

### 4.6. Gene Expression Analysis

For RT-qPCR, 1 μg of purified total RNA was used for first-strand cDNA synthesis with HiScript Q RT SuperMix for qPCR (Vazyme, China). RT-qPCR was performed in an ABI 7500 Real-Time PCR System (Applied Biosystems, USA). The PCR products were loaded on 1% agarose gels and checked after staining with ethidium bromide. Each experiment was replicated three times. The comparative Ct method was applied [53]. The sequences of the specific primers are listed in Supplementary Table S3.

### 4.7. Statistical Analyses

The Student’s *t*-test was used to determine statistical significance between the two groups. 

## 5. Conclusions

In this study, we characterized *fea5*, a novel fasciated ear mutant. *fea5* plants develop thick and flat ears with disordered and increased numbers of kernel rows in the middle of the ear. Diameter and kernel weight of *fea5* ears were also significantly greater than those of the control. By integrating BSA-seq, RNA-seq, and phytohormone quantification, we demonstrated that the fasciated ear phenotype is likely the result of a recessive mutation in *Zm00001d048841*. DEGs identified in the mapping interval were enriched for phytohormone signal transduction pathway genes, and consistently, we observed alterations in phytohormone profiles in *fea5* compared with the normal ears. *Zm00001d048841* was identified as the most likely candidate gene due to its differential expression between the mutant and normal ear and the association of its different haplotypes with maize kernel traits in a natural population. Functional verification of *Zm00001d048841* is needed in future studies. Our study adds to the current understanding of genetic control of maize ear and kernel row formation and provides valuable germplasm resources for genetic research and breeding of high-yield maize varieties.

## Figures and Tables

**Table 1:** Agronomic traits of *fea5*.

Trait	n	Normal	*fea5*
Sanya			
Plant height (cm)	40	181.2 ± 9.3	182.7 ± 8.5
Ear height (cm)	40	82.5 ± 5.5	81.0 ± 9.1
Number of maize leaves	40	9.9 ± 0.9	9.53 ± 0.64
Stem diameter (mm)	40	17.56 ± 1.82	17.85 ± 1.49
Tassel branch number	40	9.3 ± 1.6	8.8 ± 1.88
Ear diameter (mm)	40	36.2 ± 3.0	53.9 ± 2.9 ***
Ear length (mm)	40	110.1 ± 11.4	105.7 ± 14.8
Ear kernel weight (g)	40	75.25 ± 5.29	80.25 ± 3.56 **
Zhengzhou			
Plant height (cm)	40	186.0 ± 5.2	187.3 ± 8.4
Ear height (cm)	40	88.5 ± 4.0	87.2 ± 4.0
Number of maize leaves	40	10.8 ± 0.8	10.6 ± 1.5
Stem diameter (mm)	40	19.9 ± 2.5	18.9 ± 1.9
Tassel branch number	40	9.1 ± 1.0	8.9 ± 1.3
Ear diameter (mm)	40	37.1 ± 1.7	54.8 ± 1.6 ***
Ear length (mm)	40	121.0 ± 9.2	118.3 ± 16.2
Ear kernel weight (g)	40	78.25 ± 4.36	81.25 ± 2.78 *
Asterisks indicate significant differences: * *p* < 0.05, ** *p* < 0.01, *** *p* < 0.001.			

**Table 2:** Key DEGs identified between normal and *fea5* plants involved in phytohormone signal transduction from the transcriptomic analysis.

Plant Hormone Signal Transduction	Gene ID	Normal-FPKM	*fea5*-FPKM	logFC	FDR	Description
ABA signal transduction	Zm00001d018178	6.31	23.28	1.97	1.81 × 10^−4^	ABSCISIC ACID-INSENSITIVE 5-like protein 5
	Zm00001d042721	15.53	44.24	1.60	8.36 × 10^−5^	G-box-binding factor 4
	Zm00001d020938	1.15	0.30	−1.82	4.26 × 10^−2^	bZIP transcription factor family protein
	Zm00001d047220	58.29	23.03	−1.26	5.44 × 10^−5^	Serine/threonine-protein kinase SRK2C
IAA signal transduction	Zm00001d030310	37.73	8.31	−2.17	1.57 × 10^−5^	auxin import carrier1
	Zm00001d013130	2.29	0.45	−2.41	2.36 × 10^−3^	Transcription factor PIF4
	Zm00001d034298	8.34	3.66	−1.11	2.54 × 10^−3^	Transcription factor PIF4
	Zm00001d008749	44.85	9.46	−2.13	7.64 × 10^−10^	barren inflorescence1
	Zm00001d049715	2.57	6.99	1.53	3.29 × 10^−2^	IAA25-auxin-responsive Aux/IAA family member
	Zm00001d026308	0.84	24.62	4.89	5.26 × 10^−5^	Auxin-responsive protein SAUR71
	Zm00001d015354	5.71	29.55	2.49	4.65 × 10^−3^	Auxin-responsive protein SAUR71
	Zm00001d041418	2.47	12.93	2.49	1.76 × 10^−7^	Auxin-responsive protein IAA4
CK signal transduction	Zm00001d018380	35.94	16.49	−1.02	2.94 × 10^−4^	Two-component response regulator ARR12
	Zm00001d033786	28.65	9.59	−1.48	1.06 × 10^−11^	histidine kinase4
ETH signal transduction	Zm00001d031445	4.82	2.00	−1.15	4.42 × 10^−3^	ETHYLENE INSENSITIVE 3-like 3 protein
	Zm00001d003451	11.63	0.15	−7.90	2.58 × 10^−15^	ETHYLENE INSENSITIVE 3-like 5 protein
	Zm00001d053642	58.61	15.63	−1.77	1.75 × 10^−3^	EIN3-binding F-box protein 1
	Zm00001d000408	39.65	10.01	−1.73	2.92 × 10^−3^	EIN3-binding F-box protein 1
	Zm00001d036880	76.05	17.43	−1.99	1.75 × 10^−6^	EIN3-binding F-box protein 1
GA signal transduction	Zm00001d052126	3.46	1.04	−2.11	1.31 × 10^−2^	SLR1
	Zm00001d002410	159.47	455.45	1.57	1.42 × 10^−2^	Probable xyloglucan endotransglucosylase/hydrolase protein 21
JA signal transduction	Zm00001d027900	4.58	42.21	3.22	8.84 × 10^−4^	Protein TIFY 10B
	Zm00001d014253	35.24	199.52	2.39	8.32 × 10^−4^	ZIM motif family protein
	Zm00001d042833	83.14	35.50	−1.13	5.52 × 10^−4^	Coronatine-insensitive protein 1
	Zm00001d033049	5.66	63.32	3.47	4.48 × 10^−3^	ZIM motif family protein
	Zm00001d011377	9.69	3.75	−1.22	1.77 × 10^−3^	Jasmonic acid-amido synthetase JAR1
	Zm00001d034944	7.29	15.10	1.15	3.25 × 10^−4^	Regulatory protein NPR3
	Zm00001d014249	1.19	12.02	3.37	8.63 × 10^−4^	ZIM-transcription factor 29
	Zm00001d034536	34.87	130.87	1.88	3.62 × 10^−2^	Protein TIFY 10B
	Zm00001d020614	20.72	118.89	2.55	3.29 × 10^−4^	ZIM-transcription factor 28
	Zm00001d033050	30.08	235.53	2.98	2.46 × 10^−4^	ZIM motif family protein
	Zm00001d047017	11.78	33.06	1.56	1.68 × 10^−2^	Putative HLH DNA-binding domain superfamily protein

## Data Availability

The data presented in this study are available in this article and Supplementary Materials.

## Supplementary Materials

The following supporting information can be downloaded at: https://www.mdpi.com/article/10.3390/ijms24021182/s1.
