# Supplementary material for: Integrating BSA-Seq with RNA-Seq Reveals a Novel Fasciated Ear5 Mutant in Maize
Source: Int J Mol Sci. 2023 Jan 7;24(2):1182. doi: 10.3390/ijms24021182 (PMC9867142; doi:10.3390/ijms24021182)
Supplement: Supplementary file 1 [file ijms-24-01182-s001.zip › supplementary figureS1.pptx]

## Slide 1
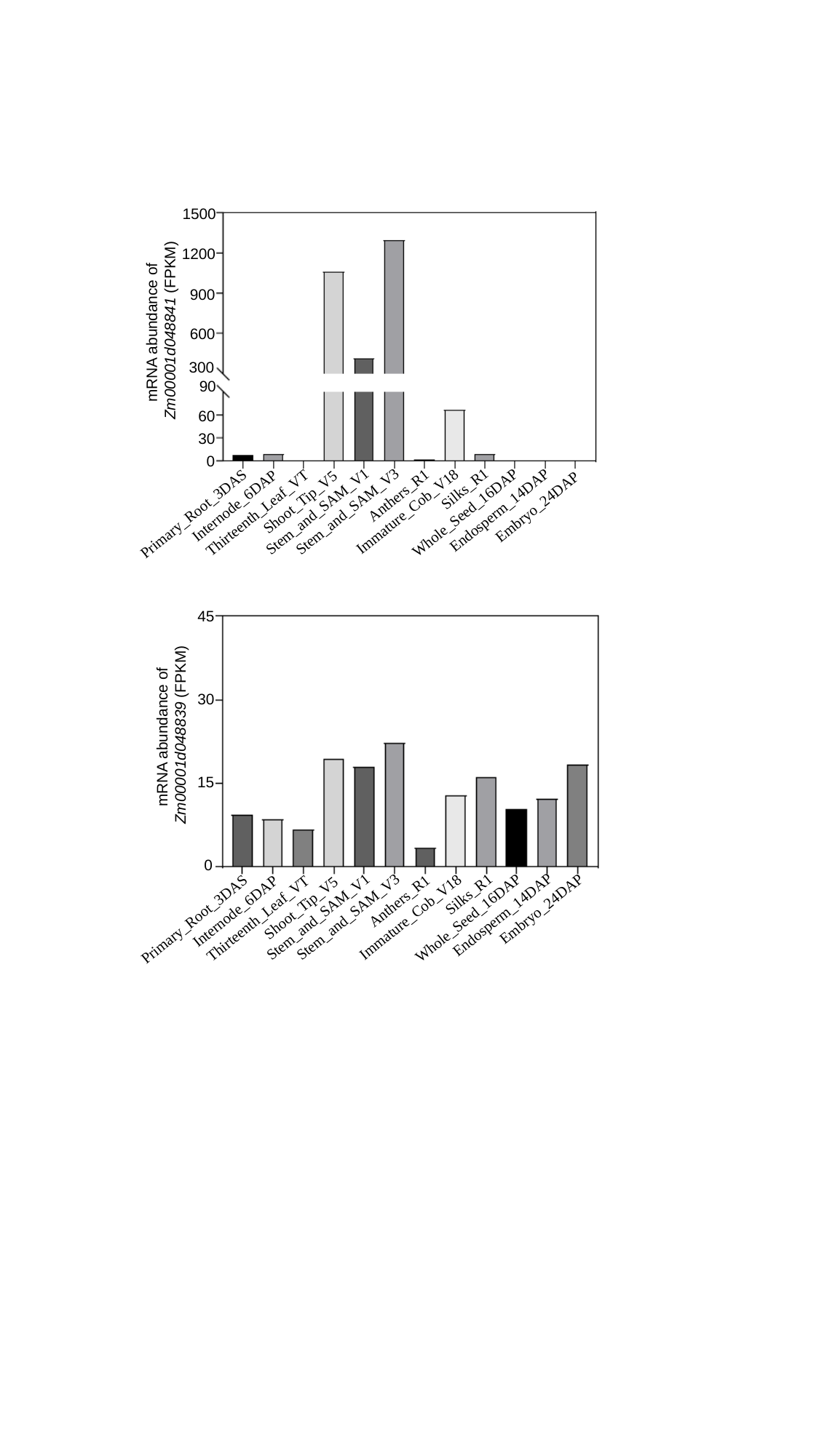

1500
1200
900
mRNA abundance of
Zm00001d048841 (FPKM)
600
300
90
60
30
0
Silks_R1
Anthers_R1
Shoot_Tip_V5
Internode_6DAP
Embryo_24DAP
Endosperm_14DAP
Stem_and_SAM_V1
Stem_and_SAM_V3
Immature_Cob_V18
Whole_Seed_16DAP
Thirteenth_Leaf_VT
Primary_Root_3DAS
45
30
mRNA abundance of
Zm00001d048839 (FPKM)
15
0
Silks_R1
Anthers_R1
Shoot_Tip_V5
Embryo_24DAP
Internode_6DAP
Endosperm_14DAP
Stem_and_SAM_V1
Stem_and_SAM_V3
Immature_Cob_V18
Whole_Seed_16DAP
Thirteenth_Leaf_VT
Primary_Root_3DAS
