# Supplementary figures and images for: Integrating BSA-Seq with RNA-Seq Reveals a Novel Fasciated Ear5 Mutant in Maize
Source: Int J Mol Sci. 2023 Jan 7;24(2):1182. doi: 10.3390/ijms24021182 (PMC9867142; doi:10.3390/ijms24021182)

## Slide 1
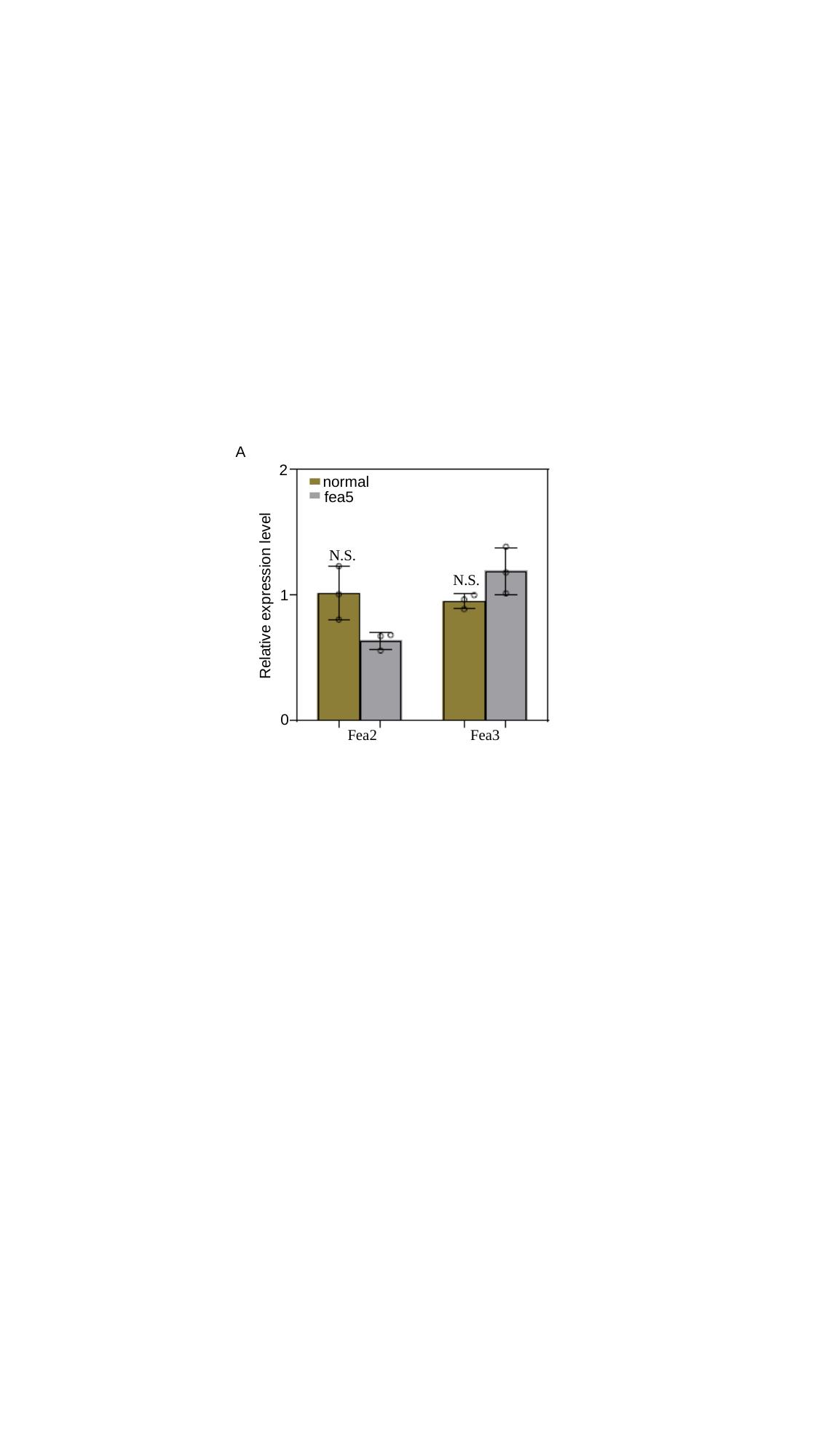

A
2
normal
fea5
Relative expression level
1
0
Fea2
Fea3
N.S.
N.S.

## Slide 2
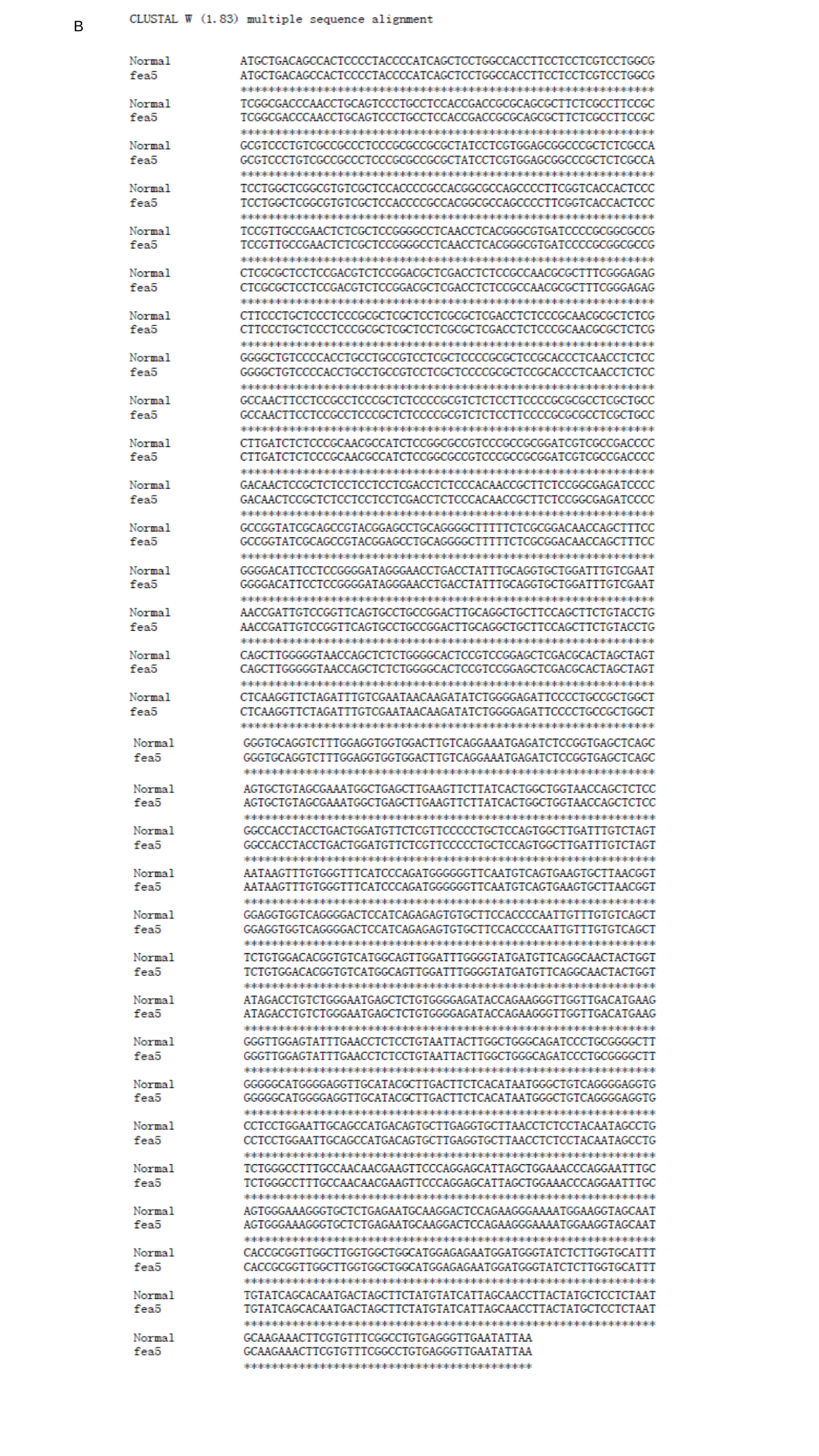

B

## Slide 3
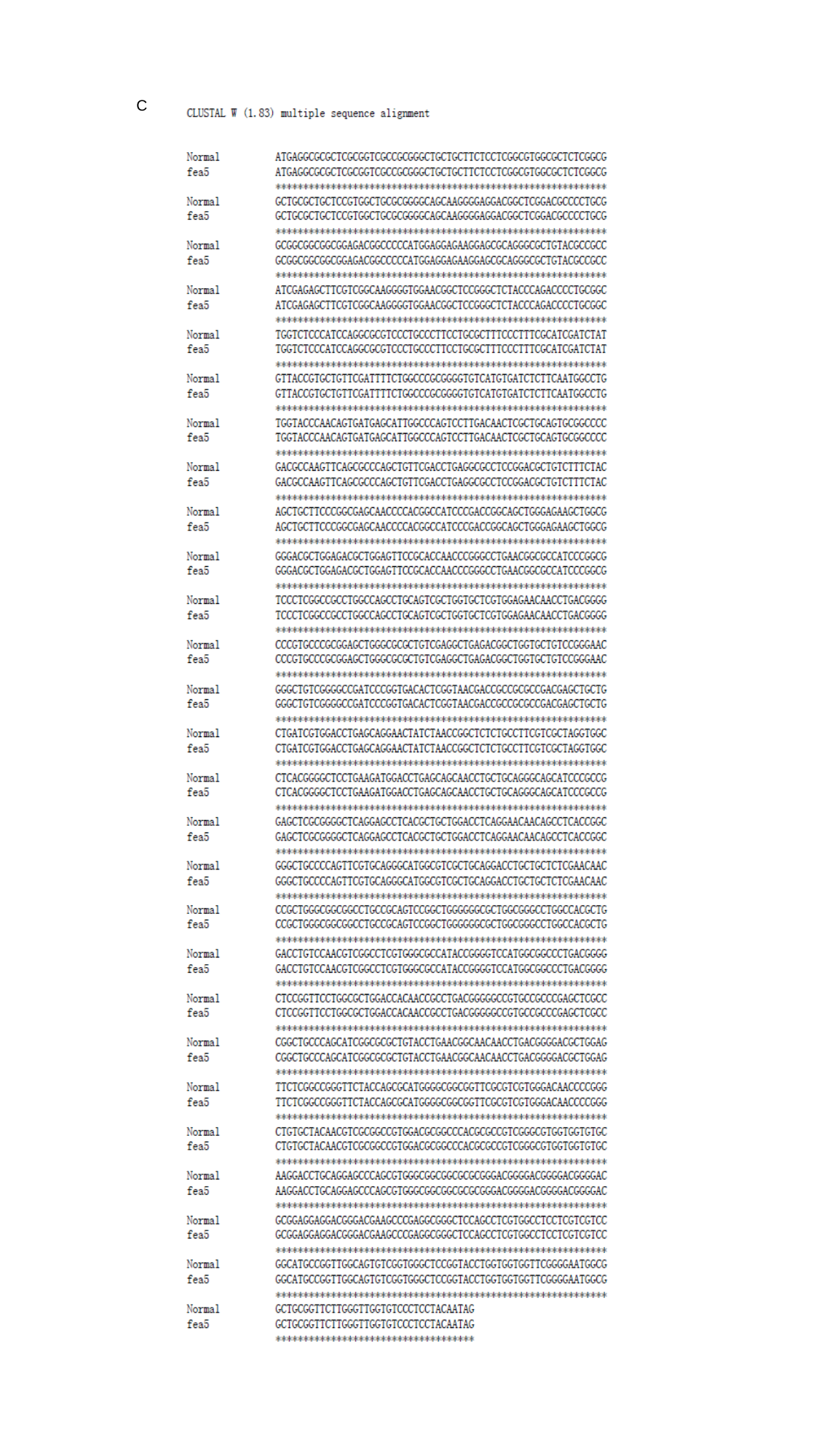

C

Supplement: Supplementary file 1 [file ijms-24-01182-s001.zip › supplementary figureS2.pptx]
